# The multidomain flavodiiron protein from *Clostridium difficile* 630 is an NADH:oxygen oxidoreductase

**DOI:** 10.1038/s41598-018-28453-3

**Published:** 2018-07-05

**Authors:** Filipe Folgosa, Maria C. Martins, Miguel Teixeira

**Affiliations:** Instituto de Tecnologia Química e Biológica António Xavier, Universidade Nova de Lisboa, Av. da República, 2780-157 Oeiras, Portugal

## Abstract

Flavodiiron proteins (FDPs) are enzymes with a minimal core of two domains: a metallo-β-lactamase-like, harbouring a diiron center, and a flavodoxin, FMN containing, domains. FDPs are O_2_ or NO reducing enzymes; for many pathogens, they help mitigate the NO produced by the immune system of the host, and aid survival during fluctuating concentrations concentrations of oxygen. FDPs have a mosaic structure, being predicted to contain multiple extra domains. *Clostridium difficile*, a threatening human pathogen, encodes two FDPs: one with the two canonical domains, and another with a larger polypeptide chain of 843 amino acids, CD1623, with two extra domains, predicted to be a short-rubredoxin-like and an NAD(P)H:rubredoxin oxidoreductase. This multi-domain protein is the most complex FDP characterized thus far. Each of the predicted domains was characterized and the presence of the predicted cofactors confirmed by biochemical and spectroscopic analysis. Results show that this protein operates as a standalone FDP, receiving electrons directly from NADH, and reducing oxygen to water, precluding the need for extra partners. CD1623 displayed negligible NO reductase activity, and is thus considered an oxygen selective FDP, that may contribute to the survival of *C. difficile* in the human gut and in the environment.

## Introduction

Flavodiiron proteins (FDPs) are widely spread in nature, namely in anaerobes and in oxygenic phototrophs, being endowed with nitric oxide (NO) and/or oxygen reductase activity (recently reviewed^1^). FDPs play a key role in the protection of living cells against these molecules and, in regard to NO, as the response of pathogens against the innate immune system, whose cells produce NO upon infection to combat invading pathogens. FDPs also protect against oxidative stress, by directly reducing the potentially toxic oxygen molecule to water, thus avoiding the formation of reactive oxygen species, and by directly eliminating the O_2_ molecule itself. FDPs have been found to be particularly important in anaerobes, that, even if only transiently, may be exposed to toxic oxygen levels^2–7^.

FDPs are soluble enzymes, cytoplasmatic, characterized by the presence of a two-domain core, that gave origin to their name: a metallo-β-lactamase-like domain, harbouring a diiron catalytic center, where substrate reduction occurs, followed by a flavodoxin-like domain, containing a flavin mononucleotide (FMN); the minimal functional unit is a head-to tail homodimer, in which one of the oxygens of the carboxylate sidechain from a monodentate iron glutamate ligand from one monomer is at ca 4 Å from the C4 methyl group of the FMN from the other monomer, assuring fast electron transfer between the two redox centers. FDPs are completely distinct from membrane bound respiratory oxygen or NO reductases, of the heme-copper superfamily, as well as from the cytochrome *bd* or the alternative diiron containing oxygen reductases^8^. While both types of enzymes use protons for the chemical reaction of O_2_ reduction into two molecules of H_2_O, FDPs don´t conserve energy. This difference extends also to the type of electron donors: while the respiratory enzymes receive electrons from membrane-associated quinols or soluble electron carriers (as HiPIPs, cytochromes *c* or copper proteins^8^). FDPs receive electrons in general from rubredoxins, NADH/FAD dependent oxidoreductases (e.g.,^1^) or, in methanogens, from the F_430_ coenzyme^9^. While some organisms contain both respiratory oxygen reductases and FDPs, many others apparently rely only on FDPs to eliminate oxygen.

FDPs are a prototype of modular enzymes that may harbour up to three additional domains, with putative extra redox centers (rubredoxins, flavins, Fe/S clusters) besides the two common core domains. These extra domains have been predicted to exist on the basis of amino acid sequence analysis and structural predictions^10,11^. One of these complex FDPs is a putative enzyme from *Clostridium* (*C*.) *difficile* 630, a virulent and multidrug-resistant strain, recently placed in the Peptostreptococcaceae family, within the Clostridia. *C. difficile* is a spore-forming Gram-positive anaerobic bacterium, present in the mammalian intestinal tract, but also found in soil and aquatic environments. It is the leading cause of infectious diarrhoea among patients in hospitals worldwide, causing diseases ranging from antibiotic-associated diarrhoea to life-threatening pseudomembranous colitis. *C. difficile* is now considered to be one of the most important causes of health care-associated infections, and most importantly infections are also emerging in the community and in animals used for food, i.e., they are no longer considered simply as a result of antibiotic therapy; aggressive, hypervirulent strains have appeared in recent years^12–14^

**Figure 1:**
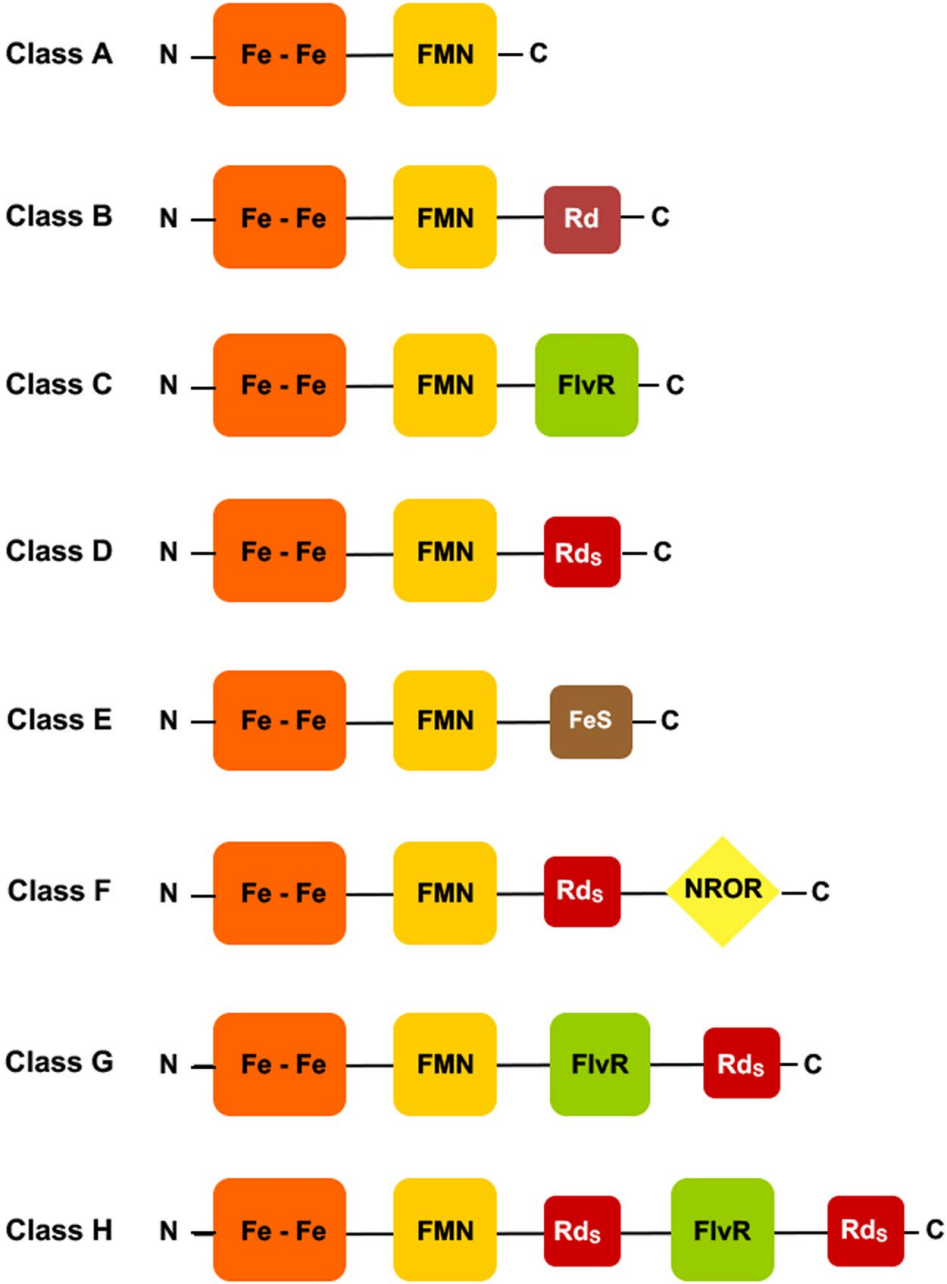
Schematic representation of the several Classes of FDPs^11^. Fe-Fe – metallo-β-lactamase domain; FMN – flavodoxin domain; Rd - canonical rubredoxin domain; Rd_S_ - short rubredoxin domain; NROR – NADH:rubredoxin oxidoreductase domain; FlvR – flavin reductase-like domain.

Albeit the gut is considered to be quite anaerobic, oxygen concentrations in it may fluctuate over time^15^, which presents a challenge to an oxygen sensitive bacterium as *C. difficile*. In fact, these strictly anaerobic bacteria are in contact with aerobic host cells, some of which also produce NO, so that enzymatic systems to detoxify oxygen and/or NO are vital for their survival within the host. However, very little is known about the response of *C. difficile* to oxidative stress as well as to NO and reactive nitrogen species, produced in the gut and, in particular, by the host immune system upon infection. The genome of *C. difficile* 630^16^ encodes for proteins considered to be involved in this response, including a Mn-SOD, a 2Fe-superoxide reductase (desulfoferrodoxin), manganese catalases, reverse rubrerythrins and two flavodiiron proteins. These two FDPs, encoded by the *cd1157* and *cd1623* genes are annotated as anaerobic nitric oxide reductase and NADH oxidoreductase, respectively, and correspond indeed to Class A and Class F FDPs (Fig. 1)^11^. Both *cd1157* and *cd1623* are upregulated upon exposure of *C. difficile* cultures to oxygen^17^, and are under the control of the general stress regulator σ^B ^^18^. Moreover, a *sigB* mutant was clearly more sensitive to oxidative and nitrosative stresses as compared to the wild type strain, suggesting that those two proteins may play a role in these responses.

To contribute to the understanding of the stress responses of the important pathogen *C. difficile*, and at the same time functionally characterize one of the predicted most complex FDPs, we here present a thorough characterization of the *cd1623* gene product and show it is a bona fide standalone NADH:oxygen oxidoreductase.

## Results

### Amino acid sequence analysis

**Figure 2:**
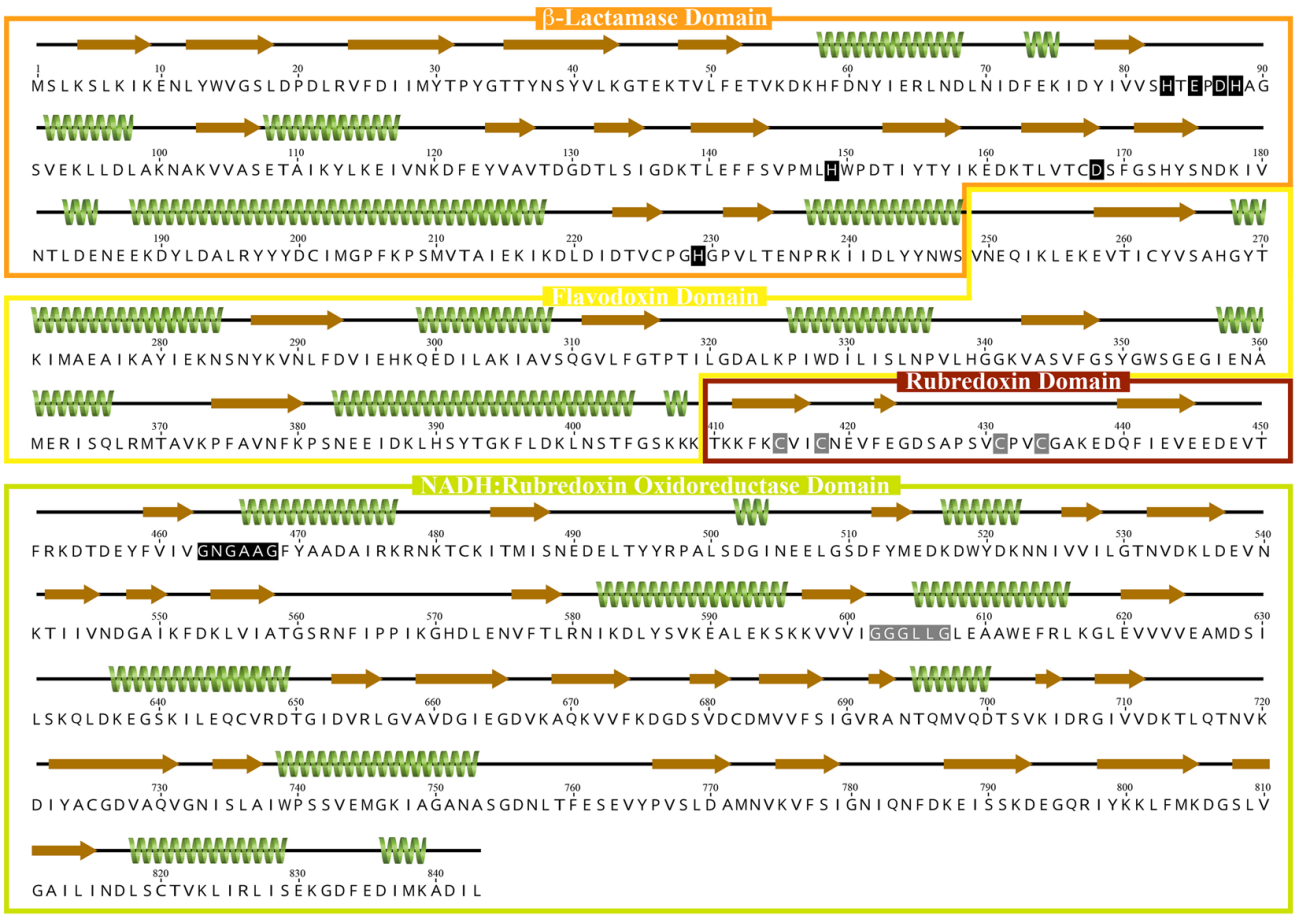
Schematic representation of FDP_F protein. Amino acid sequence of FDP_F, with predicted alfa helixes and beta strands represented as green coils and as brown arrows, respectively. Secondary structure prediction was performed with Phyre2^26^. The four domains predicted by amino acid sequence analysis are highlighted in the coloured boxes; metallo-β-lactamase domain in orange, Flavodoxin domain in yellow, Rubredoxin domain in dark red and the NADH:Rubredoxin oxidoreductase domain in green. The ligands for the di-iron and iron centers in the β-Lactamase and Rubredoxin domains are marked with black and grey boxes, respectively; the two glycine motifs of the NADH:rubredoxin oxidoreductase domain are highlighted with black (FAD binding) and grey (NADH binding) boxes.

The *cd1623* gene encodes for a very large protein, with a total of 843 amino acid residues (Fig. 2). The first ca 400 residues correspond to the flavodiiron core, comprised of the two shared consensus domains: the metallo-β-lactamase like and the small flavodoxin like ones, which exhibit 21–28% identity and 41–50% similarity with the FDPs for which crystal structures are available (Supplementary Fig. S1). *C. difficile* genome encodes for another FDP (CD1157), of the Class A, i.e., having only the two core domains, which has 39% identity and 63% similarity with FDP_F, comparing only those domains. In both *C. difficile* enzymes, the amino acid residues that bind the diiron site of FDPs are fully conserved (His83, Glu85, Asp87, His88, His149, Asp168, His229, CD1623 numbering used in the article). Following the FDP core there is a small domain of about 40 amino acids that contains two CysxxCys motifs, (Cys415/Cys418, Cys431/Cys433) separated by 12 residues. This domain is similar to the short-spaced rubredoxin (Rd) domain of classical rubrerythrins^19^, enzymes with a four-helix bundle architecture harbouring a diiron catalytic site, and an electron-transfer rubredoxin domain, binding a FeCys_4_ center^20^; this short Rd domain, not restricted to rubrerythrins nor common to the whole rubrerythrin family (see, e.g.,^19^), differs from canonical rubredoxins by the small spacing between the two cysteines motifs, ca 12 as compared with ca 30–40^20,21^. The fourth domain of this FDP (residues ~450–843) is identified by the Conserved Domain Search Service at NCBI^22^ as belonging to the NirB superfamily, which comprises a rather vast group of NADH/FAD dependent oxidoreductases, including, for example, the NADH:rubredoxin oxidoreductases from *C. acetobutylicum* and *Pseudomonas aeruginosa*^23,24^, as well as the NADH:flavorubredoxin (NO reductase) oxidoreductase from *E. coli*^25^. Amino acid sequence alignments between these proteins show low residue conservation, excepting for the nucleotide binding motifs. Nevertheless, the structure for this domain was predicted with high confidence at the Protein Homology/analogy Recognition Engine (Phyre2^26^) using as templates, among others, the structures of rubredoxin reductases, revealing highly similar tri-dimensional architectures (data not shown). Besides the overall structure conservation, this FDP_F domain shares 16–23% amino acid identity and 48–57% similarity (Supplementary Fig. S2) with those enzymes, including the two nucleotide binding motifs, GxGxxG (Gly463, Gly465,Gly468, and Gly602, Gly604, Gly607, Fig. 2), of which the first (not strictly conserved among the templates used) is involved in the binding of FAD and the second of NADH (e.g.,^23,24^). This data suggests that CD1623 is a standalone enzyme capable of receiving electrons directly from NAD(P)H without the participation of external protein partners, as will be shown below.

The presence of these four domains in this FDP identifies it as a Class F enzyme^11^, one of the most complex predicted FDPs, thus far uncharacterized, and we will hereafter designate it as FDP_F (and the C-terminal domains, deprived of the FDP core, residues 401–843, as FDP_F_Cter). Class F FDPs occur mainly in *Firmicutes* (namely from the *Clostridiales* order), but are also present in distant bacterial phyla, such as in some *Fusobacteria*, *Fibrobacteres* and *Bacteroidetes*, as well as in the eukaryotic protozoa *Trichomonas vaginalis* and *Tritrichomonas foetus*^11^. These proteins share a high amino acid sequence identity among themselves (identities above 39%, similarities above 70%), and the amino acids binding the diiron and the rubredoxin-type centers, as well as the glycines of the second glycine rich motif are strictly conserved (Supplementary Fig. S3); at the first GlyxGlyxxGly motif there are a few variations, namely at the third glycine (an alanine in several sequences, as also observed for the NADH/FAD dependent oxidoreductases, see supplementary Fig. S2).

### Protein characterization

**Figure 3:**
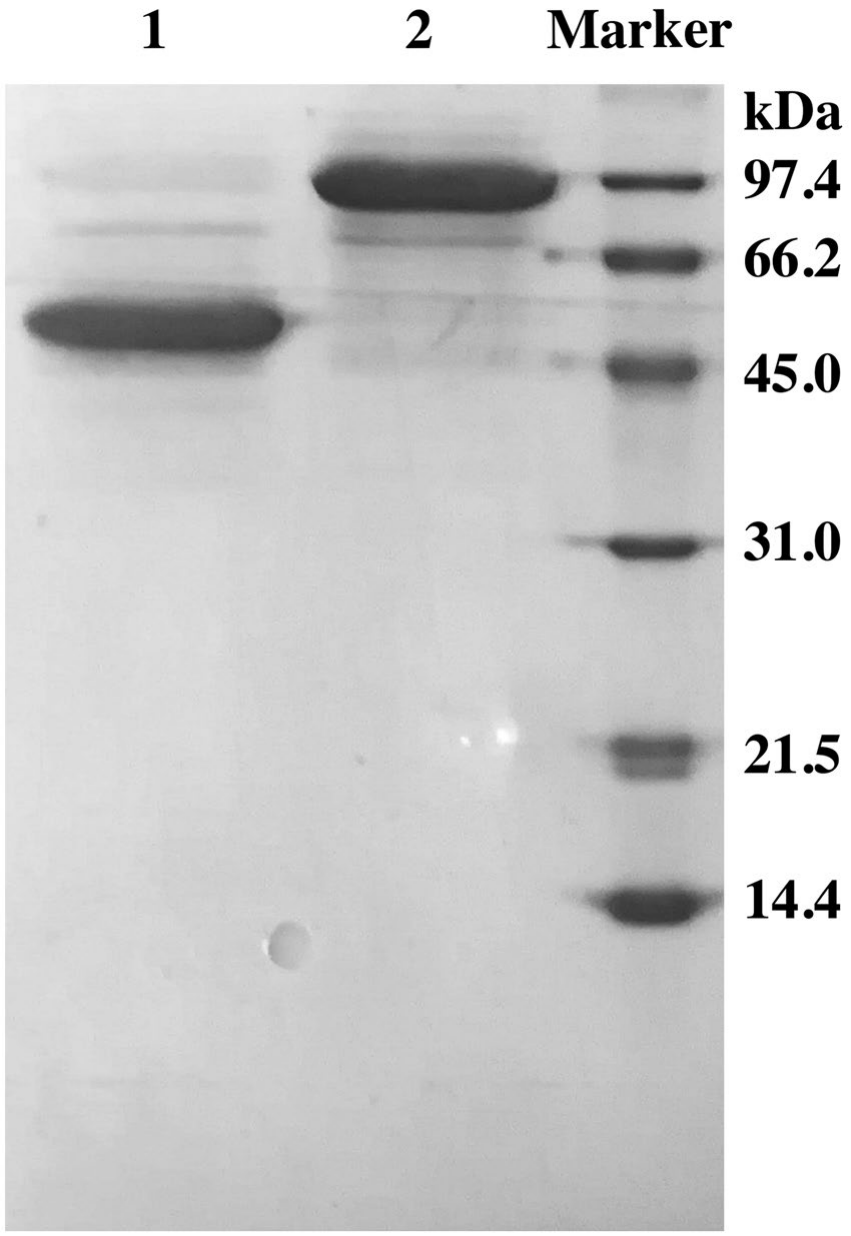
SDP-PAGE gel from FDP_F and FDP_F_Cter. Lane 1 contains FDP_F_Cter and lane 2 contains FDP_F. Low-range molecular mass markers from Bio-Rad were used as standards.

The full FDP protein (FDP_F) and its C-terminal domains (FDP_F_Cter) were successfully overexpressed in *E. coli* and purified close to homogeneity (Fig. 3). The molecular masses determined from SDS-PAGE, ~90 and 48 kDa, are in close agreement with those calculated from the respective amino acid sequences, 94.3 and 48.9 kDa. The quaternary structure in solution was analysed by size exclusion chromatography, and for both proteins a homodimeric state was determined, corresponding to the total molecular masses of 190.8 kDa (FDP_F) and 99.9 kDa (FDP_F_Cter) (data not shown). All FDPs thus far characterized appear in solution as homodimers or homotetramers (a dimer of dimers), a configuration that is essential for efficient electron transfer between the FMN of one monomer and the diiron center of the other (e.g.,^9,27–29^).

The iron content was measured by ICP-AES and a value of 1.44 Fe/monomer was obtained for FDP_F and of 0.55 Fe/monomer for FDP_F_Cter. These values are lower than expected (3 and 1, respectively), indicating that complete iron incorporation was not achieved, in spite of the iron supplementation in the growth medium. Nevertheless, no zinc was detected by ICP-AES. In fact, some FDPs have been shown to incorporate zinc, which leads to inactive enzymes. In contrast, an almost full loading of flavin was determined: 1.8 and 1.0 for FDP_F and FDP_F_Cter, respectively. By reverse phase HPLC the flavin types were identified (data not shown): while the whole protein contains both FAD and FMN, in a close 1:1 ratio, the C-terminal domain contains only FAD. This result is in concordance with what is known for FDPs, that contain FMN in the flavodoxin core domain (e.g.,^1,30,31^), while NADH:rubredoxin oxidoreductases have been reported to possess FAD^23–25^.

### Spectroscopic studies

**Figure 4:**
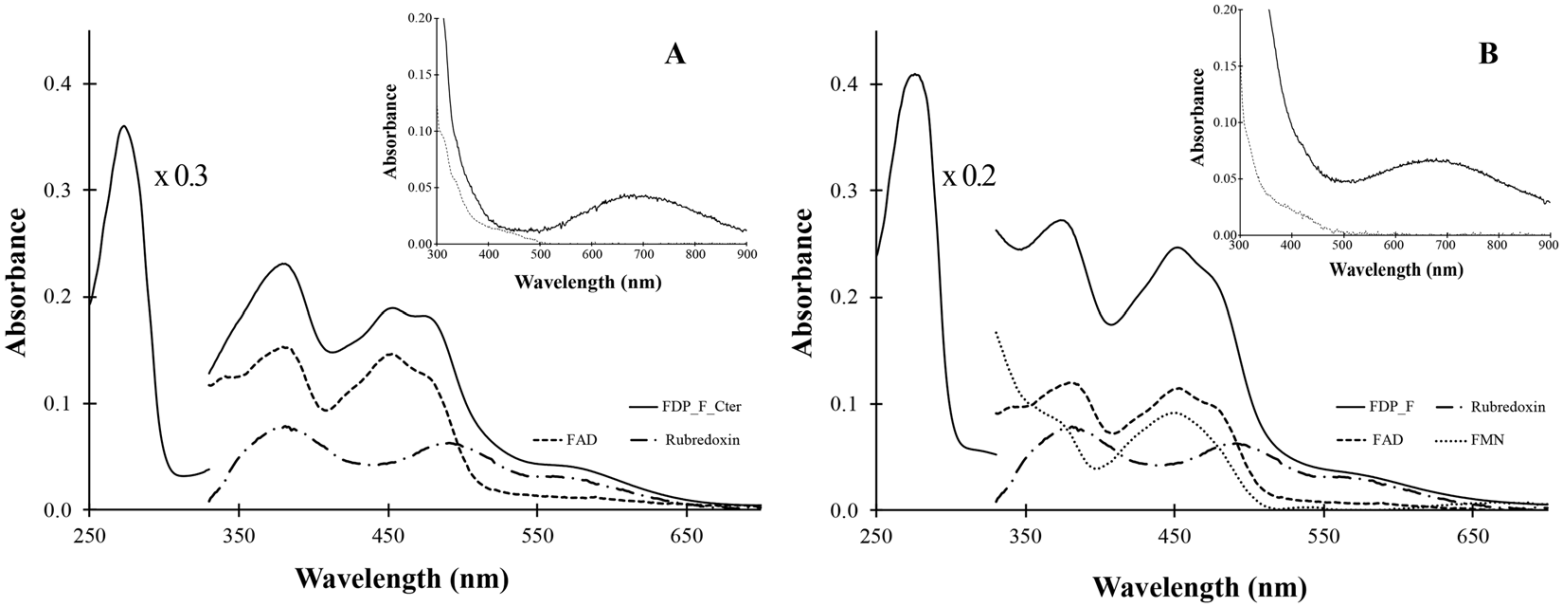
UV-Visible spectra of FDP_F and FDP_F_Cter. Panel A- Spectra of FDP_F_Cter. The black line represents the full spectrum of the as isolated (oxidized) protein, while the dashed and the dashed-dot lines correspond to the FAD and rubredoxin components. The FAD component was obtained after substoichiometric reduction with sodium dithionite, and this spectrum was used to obtain the rubredoxin component after subtraction from the spectrum of the fully oxidized protein. The insert shows the spectra after complete reduction by NADH (black line) and dithionite (dashed line). Panel B- spectra of the full FDP_F. The black line represents the full spectrum of the protein as isolated (oxidized), while the dashed and the dashed-dot lines represent the FMN, FAD and rubredoxin components. FMN component was calculated by subtraction of the full spectra minus the spectra of the rubredoxin (x1) and FAD (x0.8) components from FDP_F_Cter. The insert shows the spectra after complete reduction of the protein with NADH (black line) and dithionite (dashed line). In both spectra protein concentration was 20 µM in 50 mM Tris–HCl, pH 7.5 containing 18% glycerol.

The UV-visible absorbance spectra of as purified FDP_F and FDP_F_Cter are characteristic of complex proteins containing flavin cofactors and a rubredoxin center in the oxidized state, exhibiting several maxima between 380 and 570 nm (Fig. 4). The contribution of each of the cofactors could be obtained by deconvolution of the spectra recorded upon stepwise addition of sodium dithionite, since they have sufficiently distinct reduction potentials (see next Section), and by comparing the intact and truncated enzymes. Upon partial reduction of FDP_F_Cter (Fig. 4, Panel A), the first species to bleach was the rubredoxin component, thus enabling to obtain the spectrum of the FAD moiety, which shows a typical flavin spectrum with two major bands centered at 381 and 452 nm, this one with shoulders at 427 and 476 nm; by subtracting this spectrum from that of the fully oxidized protein, the spectrum of the rubredoxin component was obtained, with maxima at 380, 495 and ca 570 nm, characteristic of oxidized rubredoxins. The ratio of the intensities of the two spectral components is in agreement with the flavin and iron quantifications. For the full protein (Fig. 4, Panel B), it was assumed that the shape of the spectra of the C-terminal domains were unaltered, which allowed to determine the spectrum of the FMN of the flavodoxin domain, having broad bands with maxima at 374 and 451 nm, similar to those of most FDPs^32^. The component attributable to the diiron site is not observed, due to its low molar absorptivity^33^, but should have a broad maximum below 400 nm, as detected for the de-flavinated FDP from *Thermotoga maritima*^34^. Addition of excess dithionite leads to an almost complete bleaching of the spectra of both proteins in the visible region (Fig. 4, Inserts). NADH is also capable of fully reducing the proteins, under anaerobic conditions, leading in both cases to the formation of a broad band centered at 680 nm, assigned to a charge transfer complex between the reduced FAD and NAD^+^. In these reduction experiments there was no evidence for the formation of the one-electron reduced, semiquinone forms of the flavins, which should exhibit a broad absorption band at ca 600 nm (neutral form), or bands at 500/390 nm (anionic form, which so far is the one observed in FDPs).

**Figure 5:**
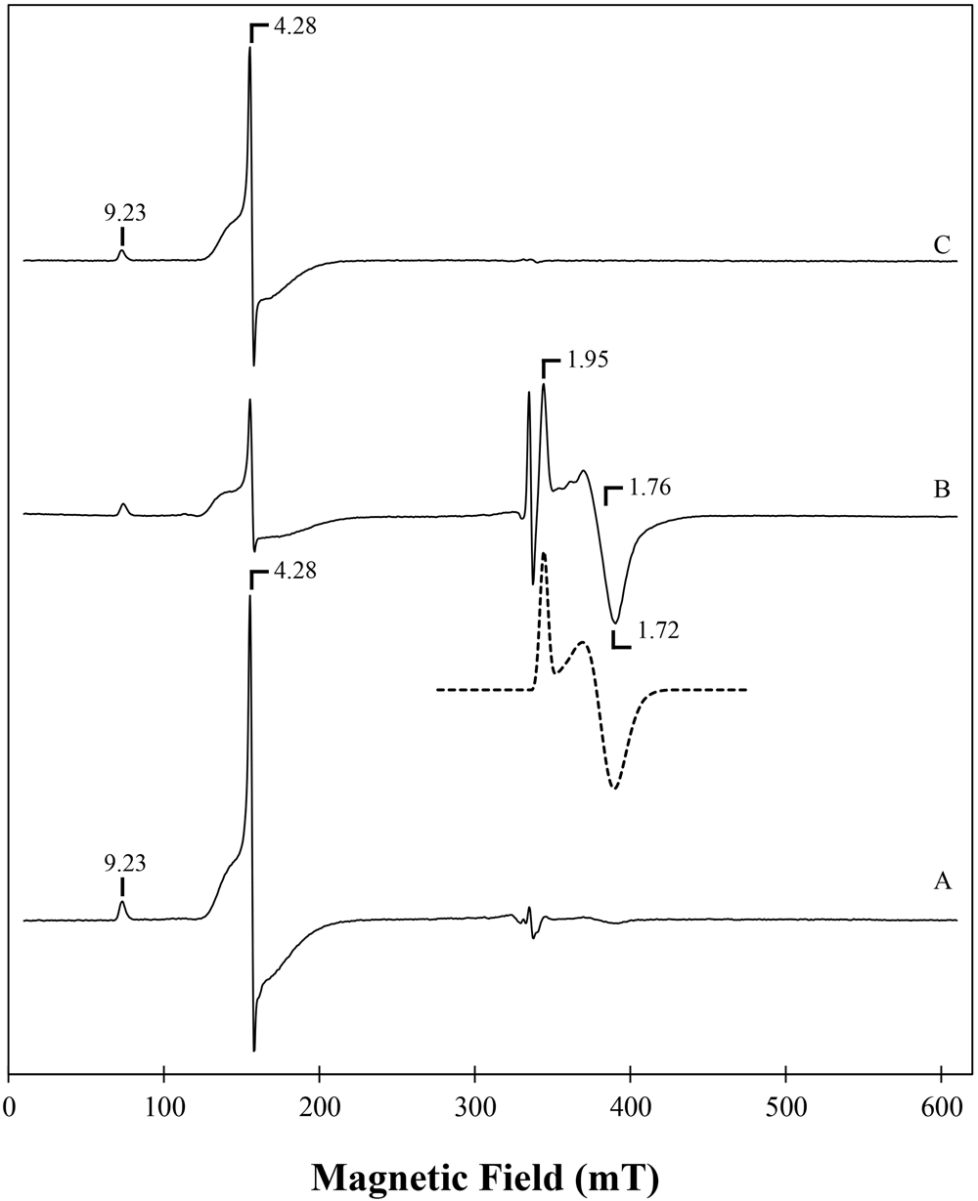
EPR spectra of FDP_F and FDP_F_Cter. Spectrum A- EPR spectrum of FDP_F (300 μM as isolated (oxidized), Spectrum B- EPR spectrum of FDP_F (sample A) after anaerobic incubation with substoichiometric menadiol. The dashed line below Spectrum B is the simulation spectrum obtained for the diiron component using *g* values of 1.96, 1.76 and 1.72. Spectrum C – EPR spectrum of FDP_F_Cter as isolated (200 μM). Temperature, 7 K; Microwave frequency, 9.39 GHz; Modulation amplitude, 1.0 mT; Microwave frequency, 2 mW.

Both FDPs were analysed by EPR spectroscopy (Fig. 5). In the as purified (oxidized) state, they exhibit at 7 K virtually identical signals at g = 4.28, and a minor resonance at g = 9.23, attributable respectively to the middle (|±3/2>) and lower (|±1/2>) doublets of a high-spin (S = 5/2) ferric center, with a rhombicity (E/D) close to 0.33, characteristic of ferric rubredoxin sites. The identity of the signals of FDP_F and FDP_F_Cter suggest that the rubredoxin sites are equivalent in the intact and truncated protein forms. In the spectrum of the full protein there is also a very low-intensity set of resonances in the g~2 region, which considerably increases in intensity upon anaerobic incubation of the enzyme sample with substoichiometric amounts of menadiol (Fig. 5b): a quasi-axial signal with all g-values below 2, at g = 1.96, 1.76 and 1.72 develops (g-values obtained by spectral simulation), which is typical of an antiferromagnetically coupled diiron site in the mixed valence form (Fe(III)-Fe(II)), with S = 1/2. This signal is similar to those detected previously in other FDPs and constitutes a fingerprint for the presence of the diiron site in these enzymes (e.g.,^4,25,35–37^). The rubredoxin site is partially reduced by menadiol, as revealed by the decrease in intensity of the g~4.3 resonances; also, a low-intensity radical signal at g = 2.00 is formed upon menadiol reduction, possibly due to the FMN moiety.

### Redox properties

**Figure 6:**
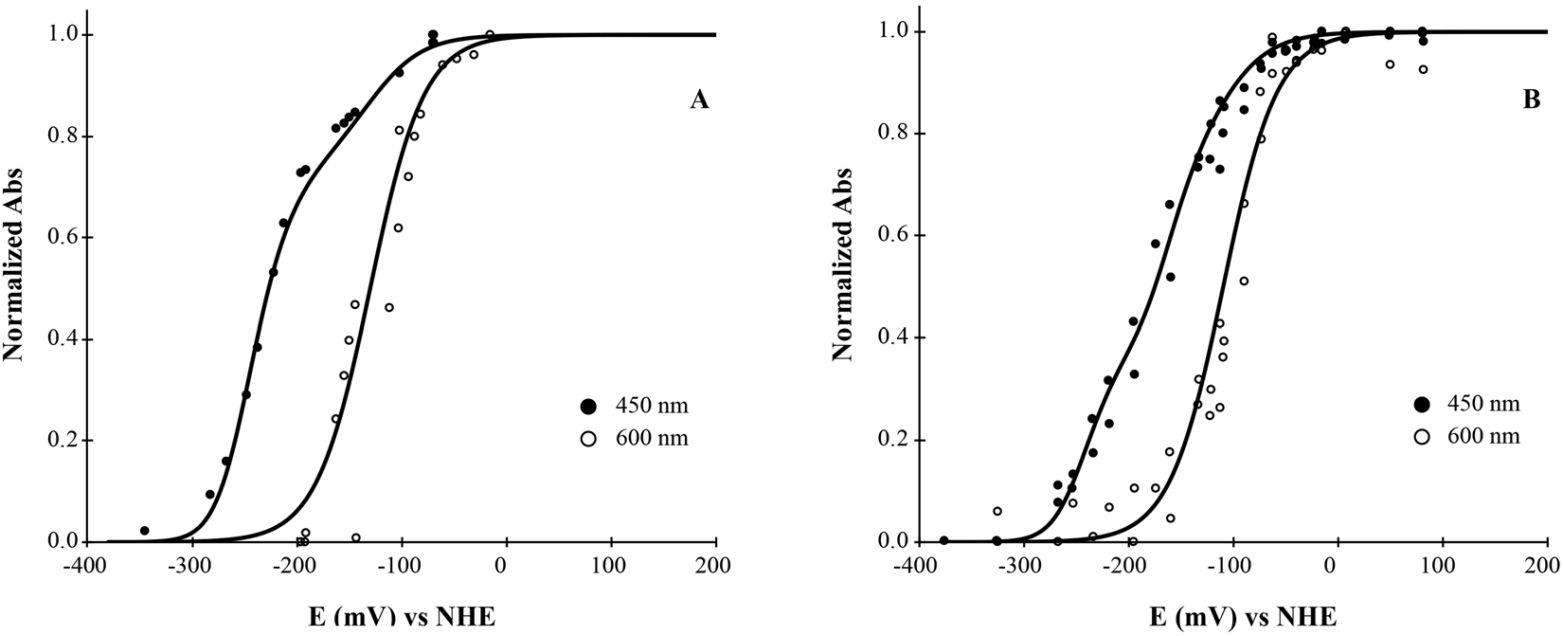
Anaerobic redox titration curves of FDP_F and FDP_F_Cter. Both panels correspond to the normalized intensities measured at the indicated wavelengths. Panel A represents the titration curve for FDP_F_Cter and Panel B for FDP_F. In both titrations protein concentration was 25 µM and the experiments were performed in 50 mM Tris–HCl, pH 7.5 containing 18% glycerol. The solid lines correspond to Nernst equations adjusted as described in the text, with the following reduction potentials: FDP_F_Cter, −130 mV (Rd), −250 mV, −250 mV (FAD); FDP_F, −110 mV (Rd), −170 mV, −170 mV (FMN), −250 mV, −250 mV (FAD).

The reduction potentials of the rubredoxin and flavin centers were obtained by redox titrations monitored by Visible spectroscopy (Fig. 6), inside an anaerobic chamber, at pH 7.5, and using for each spectral component the weights obtained from the data in Fig. 4. The rubredoxin site was monitored at 600 nm, to minimize interference from the flavins; very similar potentials were obtained for FDP_F and FDP_F_Cter: −100 ± 15 mV and −130 ± 15 mV, respectively (Fig. 6A,B). The titration was followed at other wavelengths (475, 450 nm), to monitor the flavin reduction, and taking into account the contribution of the rubredoxin center at those wavelengths. For the FDP_F_Cter the data could be well described by the sum of the Nernst equation for the Rd center plus a Nernst equation for two consecutive one-electron transitions for the FAD, yielding for this flavin two identical (within the experimental error) potentials of −250 ± 15 mV (Fig. 6A); as mentioned above for the simple chemical reductions with dithionite or NADH, in the titration there was again no evidence for the formation of a stable semiquinone form. To analyse the data for the full protein (Fig. 6B), due to the strong spectral superposition of the flavin spectra, it was assumed that the potentials for the C-terminal domain FAD would not change in FDP_F, thus decreasing the number of unknown parameters; the data was then adjusted to the sum of three weighted Nernst equations, one for the rubredoxin site, and two for two consecutive one electron transitions for the FAD and FMN moieties. Within experimental error, the data could indeed be simulated with the same potentials as those of the FDP_F_Cter, plus two identical potentials for the FMN of −170 ± 15 mV. In contrast to most other FDPs, no signature for a neutral or anionic semiquinone form of the FMN could be observed. The reason for this contrasting behaviour is presently unknown, but in part may result from the two identical reduction potentials of the FMN, which would lead to a low population of the partially reduced semiquinone form (but see also Discussion section).

### Catalytic activities

The O_2_ and NO reductase activities of the two enzymes were determined amperometrically, with modified Clark-type selective electrodes. Since the enzymes contain built-in a putative NADH oxidoreductase domain, no external redox partner was needed to perform the assays, and a large excess of NADH (5 mM) was used as the primary electron donor.

**Figure 7:**
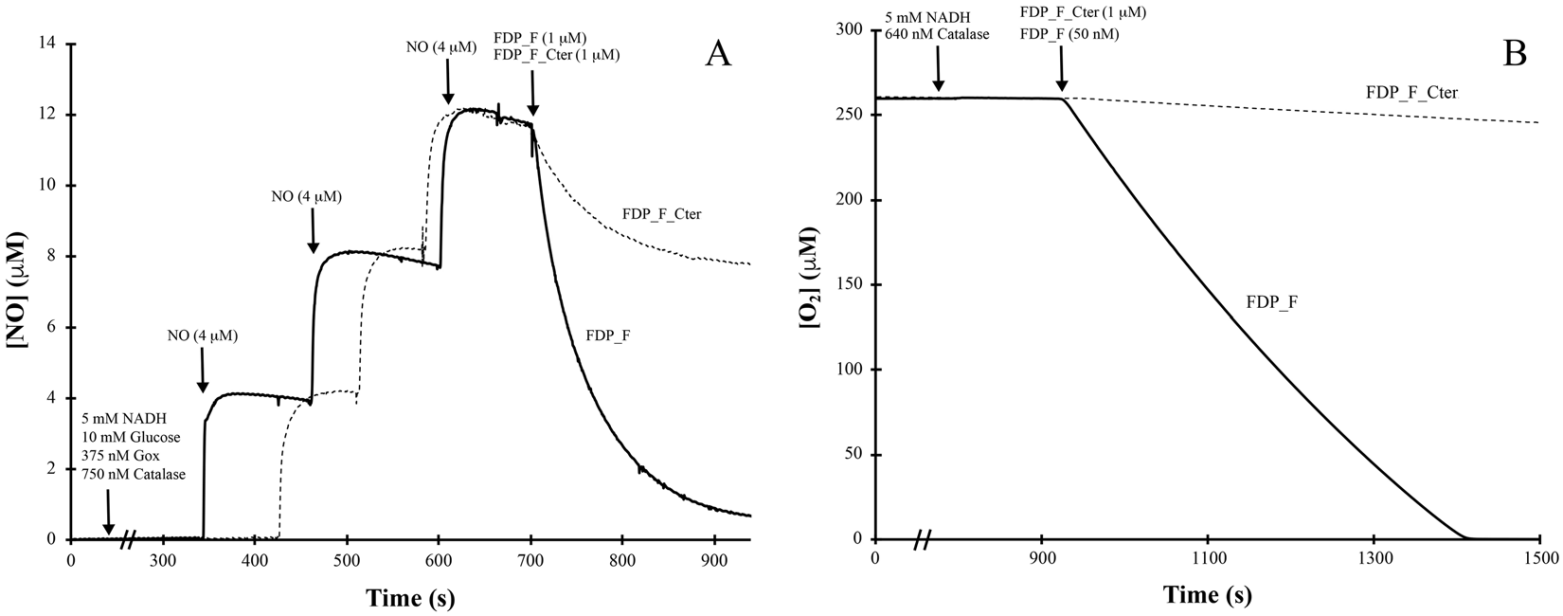
NO and O_2_ reductase activities of FDP_F and FDP_F_Cter. Panel A represents the NO reductase activity of both enzymes. Assays were performed anaerobically in a modified Clark type electrode. In both assays protein concentration was 1 µM in 50 mM Tris–HCl, pH 7.5 containing 18% glycerol. Panel B represents the O_2_ reductase activity of both enzymes. Assays were performed in a modified Clark type electrode. Protein concentrations were 50 nM and 1 µM for FDP_F and FDP_F_Cter, respectively, in 50 mM Tris–HCl, pH 7.5 containing 18% glycerol. NADH (5 mM) was used as reductant.

**Figure 8:**
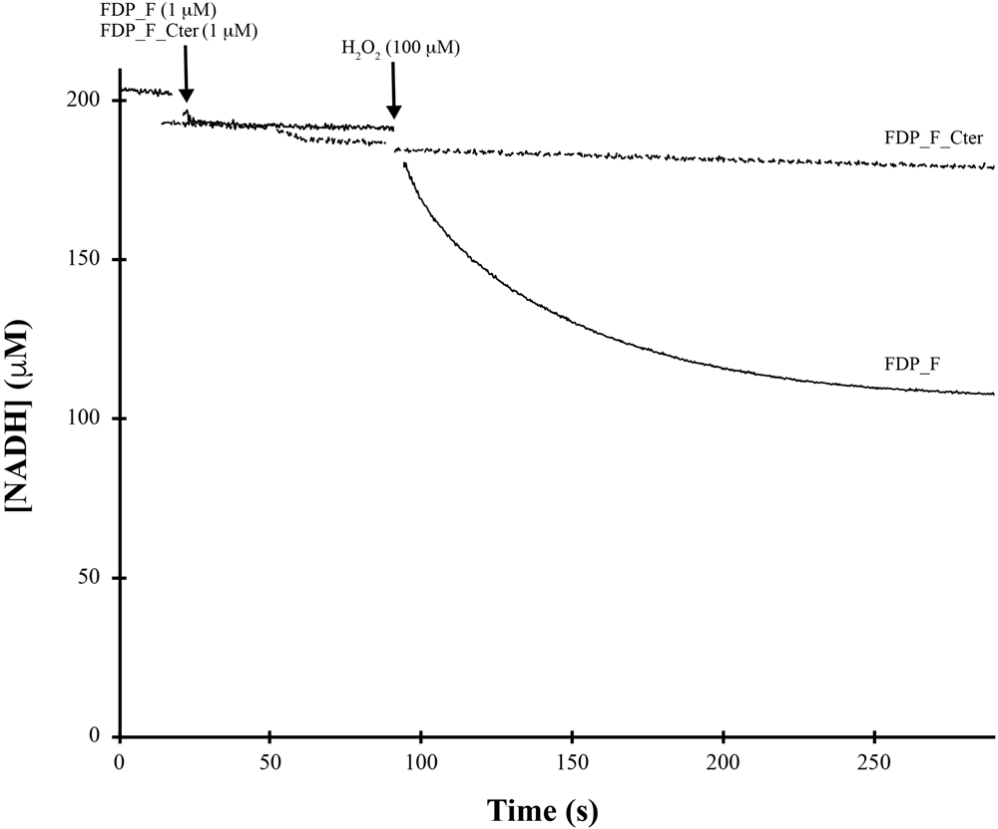
H_2_O_2_ reductase activity of FDP_F and FDP_F_Cter. H_2_O_2_ reductase activity of both enzymes. Reaction was performed anaerobically in a glove box, and monitored at 340 nm, following NADH (0.2 mM) consumption. In all assays protein concentrations were 1 µM in 50 mM Tris–HCl, pH 7.5 containing 18% glycerol.

As shown in Fig. 7, FDP_F had a quite low NO consumption activity, under anaerobic conditions, with a turnover of 0.20 ± 0.01 s^−1^; FDP_F_Cter has a much lower activity (initial rate of 0.05 ± 0.01 s^−1^), and is rapidly inactivated after a certain number of turnovers (Fig. 7A). In contrast, FDP_F displays a remarkable O_2_-reductase activity with a turnover of 16.0 ± 1.3 s^−1^ (Fig. 7B), using NADH as reductant. If NADPH is used, the reaction is much slower, 0.90 ± 0.11 s^−1^ (data not shown), indicating NADH as the preferred electron donor. The enzyme truncated form, FDP_F_Cter, exhibits a low rate of 0.03 ± 0.01 s^−1^, showing that the reductase domain has a low reactivity with oxygen. In the presence of excess NADH (5 mM) addition of catalase had no effect on the rate of O_2_ consumption by FDP_F, pointing to H_2_O (and not H_2_O_2_) as the reaction product, which was confirmed by posterior addition of catalase. This was most clearly shown by monitoring oxygen consumption at increasing concentrations of NADH (from 200 µM to 700 µM), and an NADH/O_2_ stoichiometry close to 2:1 was determined (data not shown), confirming that full reduction of oxygen to water occurs. If lower concentrations of NADH were used and catalase was omitted from the reaction mixture, inactivation of the enzyme was observed during turnover with O_2_, occurring a decrease in the rate of oxygen consumption and incomplete oxygen reduction; however, accumulation of H_2_O_2_ was not detected, examined by a possible increase in oxygen concentration after addition of catalase in the reaction chamber at the end of the reaction. Both set of observations suggest that the enzyme may have some peroxidase activity, acting also as an NADH:H_2_O_2_ oxidoreductase. This hypothesis was confirmed by measuring spectrophotometrically NADH consumption upon addition of hydrogen peroxide (Fig. 8). There is a clear H_2_O_2_ reductase activity, with a 1:1 NADH/H_2_O_2_ stoichiometry, i.e, the hydrogen peroxide is fully reduced to water; a turnover of 2.4 ± 0.5 s^−1^ was determined. Again, as seen for the other substrates tested, the FDP_F_Cter construct has a negligible H_2_O_2_ reductase activity.

## Discussion

We have isolated and characterized a new type of a complex FDP from the human pathogen *C. difficile* 630, one of the two FDPs from this organism, and which is a clear example of the modular nature of this family of enzymes. The recombinant enzyme has the cofactors that were predicted to exist by analysis of its amino acid sequence, associated with each of the four domains of Class F FDPs (Fig. 1^11^): a diiron center and an FMN, in the two core domains of FDPs, an FeCys4 center at a short-spaced rubredoxin domain (as in Classical rubrerythrins), and an FAD, at the C-terminal NADH:rubredoxin oxidoreductase domain. These FDPs are quite common in the genomes of *Clostridiales* and other Firmicutes, but are also encoded in the genomes of bacteria from other phyla, as well as in some anaerobic protozoa. Interestingly, a comparison of protozoan gene sequences with prokaryotic ones, showed that among the 15 genes that most probably resulted from lateral gene transfers from anaerobic prokaryotes (namely Clostridiales) to the protozoa, are the flavodiiron protein ones^38^; therefore, it appears likely that the two protozoa (*T. vaginalis* and *T. foetus*) that have genes encoding for Class F FDPs may have acquired them from Clostridia species.

Several FDPs have as immediate electron donors canonical rubredoxins, which in turn are reduced by NAD(P)H:rubredoxin oxidoreductases, i.e., a three-component electron transfer (eT) chain couples the NAD(P)H oxidation to the reduction of O_2_ or NO^39–42^. In *E. coli* the electron transfer chain has only two components, as the rubredoxin partner is fused to the FDP core (Class B FDPs, or flavorubredoxins, Fig. 1), and the electrons are transferred from NADH through an NADH:flavorubredoxin oxidoreductase (analogous to NADH:rubredoxin oxidoreductases)^25,43^; in organisms having Class D FDPs (which have a predicted flavodiiron core, followed by a short-spaced rubredoxin-rubrerythrin-like domain, Fig. 1^11^) the eT chain is probably similar to that of *E.coli*, as in the genomes of the organisms having these FDPs genes encoding for putative NADH:rubredoxin oxidoreductases are present (our unpublished data). Thus, Class F FDPs emerge as a fusion of the three-component eT chains, possibly having as precursor Class D enzymes, which may optimize the rate of electron transfer from the primary electron donor, NAD(P)H, to the flavodiiron catalytic core. In functional terms, Class F FDPs recall also the case of Class C enzymes (Fig. 1, present in cyanobacteria, lower eukaryotic oxygenic photosynthetic organisms and higher plants, excluding, so far, angiosperms), in the sense that both Class C and F FDPs can accept electrons directly from NADH, precluding the need of external protein partners to transfer electrons from NADH to the FDPs. In the case of the Class C enzymes, the extra module with NADH oxidase activity is similar to NADH:flavin oxidoreductases^11,44,45^, which is predicted to exist also in Classes G and H FDPs (Fig. 1^11^). But while the fusion of NAD(P)H:oxidoreductase-like domains can be easily rationalized, by allowing a faster electron transfer between NADH and the catalytic domain, the need for an additional putative electron transfer center (rubredoxin-like center), as predicted also for Class H FDPs, remains unknown; it is possible that the Rd domain may accept electrons from other thus far unidentified redox partners. The determination of the 3D structure of these enzymes will be crucial to gain insights on the role of the Rd domain. It is also interesting that in these FDPs, and in contrast with the Class B enzymes, present in proteobacteria, the rubredoxin domain is quite short and similar, in terms of spacing between the two pairs of cysteine ligands, to those of classical rubrerythrins, as above mentioned. Apart from rubrerythrins (where the Rd center acts as electron donor to the diiron site), and now the FDPs, these domains exist in quite diverse proteins, where its function and properties remain unknown (e.g., refs in^11^); it is worth pointing out that so far these short FeS domains have never been isolated as single entities.

The spectroscopic (EPR and UV-Visible) features of *C. difficile* FDP_F are characteristic of the types of centers present in FDPs, a major difference being the apparent absence of a stable semiquinone form of the FMN, which was not detected either by dithionite or NADH successive reduction of the enzyme. Interestingly, the amino acid sequence of FDP_F (as well as of all FDPs from this Class), shows that the arginine that establishes a hydrogen bond, through its main chain amino group, to the N5 of the isoalloxazine moiety of the FMN, and which has been considered responsible for the formation of the red semiquinone in the FDPs that contain this residue (e.g.,^30^), is absent in FDP_F (Fig. S1).

The reduction potentials of the flavin cofactors of FDP_F (which are essentially equal to those in the C-terminal truncated domains), are within the range of those measured for FDPs or NADH:rubredoxin oxidoreductases^25,32^. A major and unexpected difference, is the reduction potential of the rubredoxin-like domain, which has a potential of ~−110 mV, 300–400 mV lower than those in the equivalent center of rubrerythrins (+185 to + 280 mV,^46–48^); such a low value for a Rd-like center has a parallel only in the rubredoxin center of *E. coli* flavorubredoxin (−123 mV^25^), which is, nevertheless, of the “canonical” size. The high reduction potential of the Rd site in the rubrerythrins was attributed to some amino acid substitutions of aliphatic residues near the cysteines pairs in canonical rubredoxins by an asparagine and a histidine in *D. vulgaris* rubrerythrin^49^; in fact those substitutions are not observed in FDP_F, but only the determination of its 3D structure may give clues to this enormous shift in reduction potentials.

**Figure 9:**
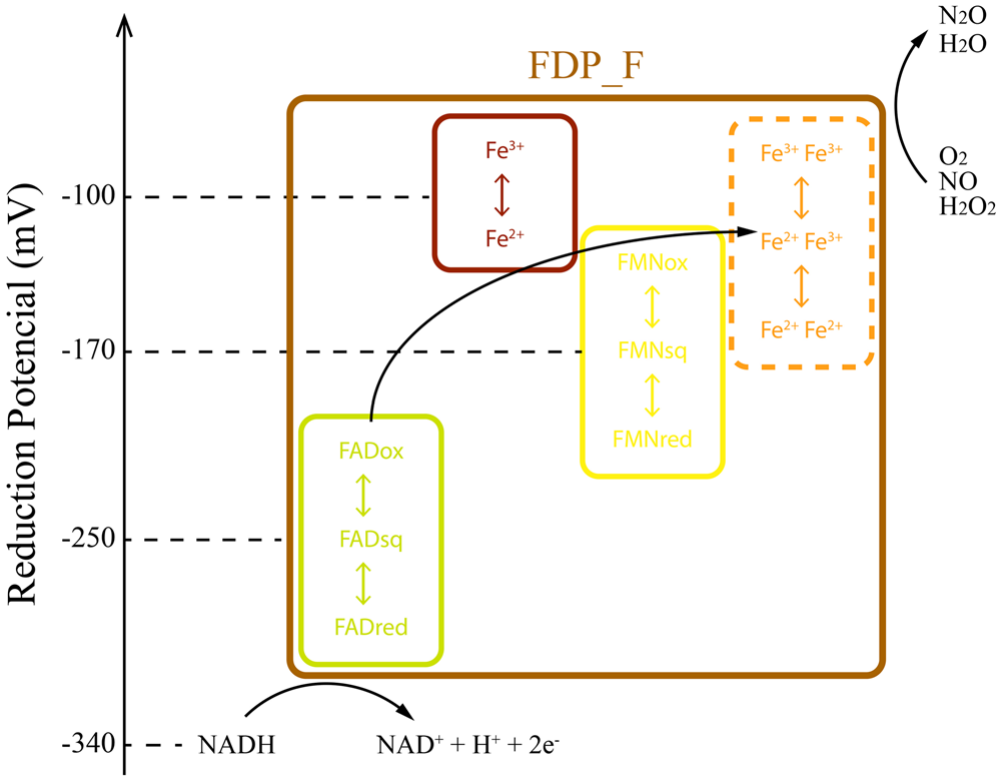
Scheme of the intramolecular electron transfer chain within *C. difficile* FDP_F. The relative position of each domain as well as an electron flow from NADH to the substrate are represented, considering the determined reduction potentials. Although not determined, the reduction potentials for the diiron center are expected to be in the range indicated, as explained in the text. Each domain is encircled with boxes of different colors: metallo-β-lactamase domain in orange, Flavodoxin domain in yellow, Rubredoxin domain in dark red and the NADH:Rubredoxin oxidoreductase domain in green. The whole protein is encircled in brown.

The reduction potential of the diiron center could not be determined in this work, but since it is partially reduced by menadiol (E_0_´ = 0 mV), it suggests that at least the potential for the first redox transition, from the full ferric form to the mixed valence one, is positive. Therefore, the reduction potentials of all FDP_F cofactors are suitable for a sequential electron transfer from NADH (the preferred electron donor, E_0_´ = −340 mV), through the flavin cofactors and the Rd site, to the catalytic diiron center where substrate reduction will occur (Fig. 9), the whole process occurring within a single polypeptide chain. The dimeric structure of FDP_F suggests that, as in other FDPs, the ultimate electron donor to the diiron site may be the flavin from the other monomer, presumably in a head to tail conformation. Again, as mentioned, the need for the Rd site is presently unknown, but it should be pointed out that the C-terminal domains do not exhibit significant activities towards any of the substrates assayed. The formation of the FAD-NAD^+^ complex may contribute to the very slow reactivity of the C-terminal domain with oxygen, as recently proposed for type II NADH:quinone oxidoreductases^50,51^.

The FDP_F from *C. difficile* is clearly an oxygen reductase, with turnovers within the range determined for other FDPs (~15–50 s^−1^), and showing an almost negligible reactivity towards NO reduction (of only 0.2 s^−1^). On the other hand, the peroxidase activity here determined is a novelty among the FDP field, but is has never been assayed with other similar enzymes; a K_M_ for this activity could not be determined, since the enzyme is inactivated at higher H_2_O_2_ concentrations (data not shown). Whether this activity has a physiological role as an additional detoxifying system in this strict anaerobe remains to be clarified, as well as whether such an activity is a general property of FDPs.

## Final Conclusions

We have demonstrated that the *C. difficile* gene *cd*1623 encodes for a large flavodiiron protein, the most complex FDP thus far characterized. Combining the analysis of the full protein and the C-terminal truncated domains it was possible to show that it contains four structural domains and several redox cofactors associated with each one, as predicted for a Class F FDP: a diiron center, an FMN (both in the FDP core), a FeCys_4_ center (in a short-space rubredoxin-like domain), and an FAD in an NADH:rubredoxin oxidoreductase like domain. The presence of these domains, with redox centers with appropriate reduction potentials to form an intramolecular electron transfer chain, allows this FDP to function as a standalone enzyme, receiving electrons directly from NADH, without the need for extra electron transfer partners. The enzyme has a clear preference for oxygen as a substrate (circa 100 times larger turnover with O_2_ than with NO), and reduces it to water. The reactivity with hydrogen peroxide, as an H_2_O_2_ reductase with formation of water, and with a non-negligible turnover (2 s^−1^) is a novelty in the field of FDPs.

*C. difficile* is a strict anaerobe, with a quite high susceptibility to oxygen. This FDP, together with the other oxidative stress proteins encoded in its genome, might confer some ability of the bacterium to tolerate small and/or fluctuating amounts of oxygen, both in the human gut and in the environment.

## Methods

### Protein expression and purification

The amino acid sequence of CD1623 from *C. difficile* 630 was retrieved from NCBI and used to synthesize its encoding gene at GenScript Inc. USA, codon optimized for expression in *E. coli*; a truncated form containing only the two C-terminal domains (residues 401 to 843) was also synthesized and codon optimized for expression in *E. coli*. The obtained recombinant proteins were named FDP_F and FDP_F_Cter. Both genes were cloned into a pET24a(+) vector at GenScript Inc. USA, utilizing the NdeI and HindIII restriction enzymes. The construct was subsequently used to transform *E. coli* BL21-Gold (DE3) competent cells (Agilent). Expression tests were conducted in order to achieve optimal protein expression conditions, namely, temperature, point of induction, IPTG concentration and growth media, which led to the choice of the best condition, now described. After this step, the best condition was used. 100 mL of Luria Bertani medium containing 50 µg/mL kanamycin was inoculated with a single colony of *E. coli* BL21-Gold (DE3) cells harbouring the pET24a(+)-FDP_F or pET24a(+)-FDP_F_Cter constructs. After growing for 16 h at 37 °C, at 150 rpm, 60 mL of this culture was used to inoculate 2 L of M9 minimal medium, in 2 L Erlenmeyer flasks, containing 50 µg/mL kanamycin and supplemented with 0.1 mM of FeSO_4_. This culture was grown at 37 °C at 150 rpm, until an optical density at 600 nm of approximately 0.4 was reached. At this point, 0.1 mM of FeSO_4_ was added, and protein expression was induced either by 50 or 500 µM of IPTG for FDP_F or FDP_F_Cter, respectively; cells were allowed to grow for 6 h at 30 °C. Cells were harvested by centrifugation, resuspended in 20 mM Tris–HCl buffer, pH 7.5 and disrupted by 3 cycles in a French press apparatus at 16000 psi (Thermo) in the presence of DNAse (Applichem). The crude extract was cleared by low-speed centrifugation at 25,000 g for 25 min and at 138,000 g for 1 h and 30 min and at 4 °C to remove cell debris and membrane fraction, respectively. The supernatant was dialyzed overnight at 4 °C against 20 mM Tris–HCl, pH 7.5 containing 18% glycerol (buffer A).

The soluble extract was then loaded onto a Q-Sepharose Fast-Flow column (160 mL, GE Healthcare) previously equilibrated with buffer A. Both proteins were eluted with a linear gradient from buffer A to 20 mM Tris–HCl, pH 7.5 containing 18% glycerol and 500 mM NaCl (buffer B), and the eluted fractions were analysed by 15% SDS-PAGE gel electrophoresis and UV–visible spectroscopy. Fractions containing the desired proteins were pooled and concentrated. FDP_F fractions were then loaded onto a size exclusion S200 column (330 mL, GE Healthcare) equilibrated with 20 mM Tris–HCl, pH 7.5 containing 18% glycerol and 150 mM NaCl. FDP_F C-ter fractions were loaded onto a Fractogel column (20 mL, Merck Millipore) previously equilibrated with buffer A and eluted with a linear gradient from buffer A to buffer B. Fractions containing pure protein were pooled and concentrated.

### Protein, metal and flavin quantification

Purified FDP_F and FDP_F_Cter samples were quantified using the Bicinchoninic Acid Kit (Thermo) and bovine serum albumin as standard. Iron content was determined by inductively coupled plasma-atomic emission spectroscopy (ICP-AES) at *Laboratório de Análises*, Chemistry Department, CQFB/REQUIMTE, FCT-UNL).

The flavin type was analysed by reverse phase HPLC, at the ITQB Small Molecule Analysis facility, using free FMN and FAD as standards. The protein samples were incubated at 100 °C for 20 min and then centrifuged at 13000 rpm for 15 min. The supernatant was passed through a 0.22 µm filter prior to injection in the HPLC column, Nova Pack C18, previously equilibrated with 0.1 M ammonium acetate/methanol (95:5, v/v) pH 6.0 and eluted with a linear gradient to 0.1 M ammonium acetate/methanol (80:20, v/v) pH 6.0. For quantification, the protein samples were incubated for 15 min with 1 M HCl and for further 30 min with 10% trichloroacetic acid at room temperature, centrifuged at 5,000 g for 20 min and neutralized with the addition of 15% of ammonium acetate. The absorbance spectrum was measured and flavin content quantified using ɛ_446nm_ = 11.1 mM^−1^.cm^−1^.

### Protein quaternary structure determination

The quaternary structure of both recombinant proteins was determined by size exclusion chromatography. FDP_F and FDP_F_Cter samples were loaded separately, at room temperature, onto a 25 mL Superdex S-200 10/300 GL column (GE Healthcare), previously equilibrated with 20 mM Tris–HCl, pH 7.5 containing 18% glycerol and 150 mM NaCl. As standards was used a mixture containing aldolase (Mm 158 kDa), ferritin (Mm 440 kDa), conalbumin (Mm 75 kDa), carbonic anhydrase (Mm 29 kDa) and cytochrome c (Mm 12.4 kDa), and dextran blue (Mm 2000 kDa) as void volume marker.

### Redox titrations

The protein´s reduction potentials were determined by UV-Visible spectroscopy using a Shimadzu UV-1603 spectrophotometer. Protein samples (25 µM in 50 mM Tris–HCl, pH 7.5 containing 18% glycerol) were anaerobically titrated inside an anaerobic chamber (Coy Lab Products) by successive additions of a buffered sodium dithionite solution in the presence of a mixture of redox mediators (1 µM of each, assay concentration): 1,2- naphtoquinone-4-sulphonic acid (E’_0_ = +215 mV), trimethylhydroquinone (E’_0_ = +115 mV), 1,4 naphtoquinone (E’_0_ = +60 mV), menadione (E’_0_ = 0 mV), plumbagin (E’_0_ = −40 mV), indigo trisulphonate (E’_0_ = −70 mV), indigo disulphonate (E’_0_ = −110 mV), 2-hydroxy-1,4-naphthoquinone (E’_0_ = −152 mV), anthraquinone-2-sulphonate (E’_0_ = −225 mV), safranine (E’_0_ = −280 mV), neutral red (E’_0_ = −325 mV) and benzyl viologen (E’_0_ = −345 mV). A combined Pt electrode (Ag/AgCl in 3.5 M KCl, as reference) was used and calibrated at 20 °C against a saturated quinhydrone solution (pH 7). All reduction potentials are quoted in relation to the standard hydrogen electrode. The experimental data was manually adjusted to appropriate Nernst equations, as described in the Results section: monoelectronic Nernst equations for the rubredoxin site, and Nernst equations for two consecutive monoelectronic transitions for the flavins, weighted according to the relative absorption intensities and concentrations of each cofactor.

### Spectroscopic Methods

UV-Visible spectra were obtained in a PerkinElmer Lambda 35 spectrophotometer. Electron Paramagnetic Resonance (EPR) spectroscopy characterization of both FDPs was performed using a Bruker EMX spectrometer equipped with an Oxford Instruments ESR-900 continuous flow helium cryostat, and a high sensitivity perpendicular mode rectangular cavity. Protein samples were prepared aerobically to final concentrations of 300 µM (FDP_F) and 200 µM (FDP_F_Cter). A partially reduced sample of FDP_F was also prepared anaerobically by incubation with 150 µM menadiol. EPR spectra were simulated using the program SpinCount^52^.

### Amperometric measurements of O2 and NO reductase activities

The O_2_ and NO reductase activities of the proteins were measured amperometrically with Clark-type electrodes selective for O_2_ (Oxygraph-2K, Oroboros Instruments, Innsbruck, Austria) or NO (ISO-NOP, World Precision Instruments, Sarasota, FL). The assays were performed in 50 mM Tris–HCl, pH 7.5 containing 18% glycerol. The O_2_ reductase activity was evaluated at 25 °C in air equilibrated buffer (~250 µM of O_2_), in the presence of 5 mM NADH or 5 mM NADPH. The reaction was initiated by addition of the respective FDP (50 nM or 1 µM, for FDP_F and FDP_F_Cter, respectively). Assays were started either in the presence or absence of catalase (640 nM, from bovine liver). The NO reductase activity was determined under anaerobic conditions in 50 mM Tris–HCl, pH 7.5 containing 18% glycerol and in the presence of the O_2_ scavenging system (10 mM glucose, 375 nM glucose oxidase and 750 nM catalase). Sequential additions of NO (up to 12 µM) were followed by addition of 5 mM NADH, and the reaction was initiated by the addition of either protein (1 µM). Stock solutions of 1.91 mM NO were prepared by saturating a degassed 50 mM Tris–HCl, pH 7.5 containing 18% glycerol buffer solution in a rubber seal capped flask with pure NO gas (Air Liquide) at 1 atm on ice: gaseous NO was flushed through a 5 mM NaOH trap to remove higher N-oxides and a second trap with deionized water to remove aerosols. After this, the solution was allowed to equilibrate at room temperature.

### Spectrophotometric measurements of the H2O2 reductase activity

The H_2_O_2_ reductase activities were anaerobically determined by UV-Visible spectroscopy, inside an anaerobic chamber (Coy Lab Products). The assays were performed in 50 mM Tris–HCl, pH 7.5 containing 18% glycerol. The reaction was monitored at 340 nm, observing the NADH consumption (ɛ_340 nm_ = 6.22 mM^−1^.cm^−1^). A mixture of buffer, 200 µM of NADH and 100 µM of H_2_O_2_ was prepared inside a cuvette, after which the reaction was initiated by the addition of either protein (1 µM).

### Amino acid sequence analysis

Amino acid sequences were retrieved from the public databases using BlastP; sequence alignments were performed by Clustal X^53^ or, when based on the crystal structures, by Modeller^54^ or Pymod2.0^55^. Secondary and tertiary structures were predicted using Phyre2^26^.

## Electronic supplementary material

Supplementary Information
