# Supplementary material for: The multidomain flavodiiron protein from Clostridium difficile 630 is an NADH:oxygen oxidoreductase
Source: Sci Rep. 2018 Jul 5;8:10164. doi: 10.1038/s41598-018-28453-3 (PMC6033852; doi:10.1038/s41598-018-28453-3)
Supplement: Supplementary file 1 — Supplementary Information [file 41598_2018_28453_MOESM1_ESM.pdf]

SUPPLEMENTARY INFORMATION

The multidomain flavodiiron protein from *Clostridium difficile* 630 is an NADH:oxygen oxidoreductase

Filipe Folgosa, Maria C. Martins and Miguel Teixeira

Includes Figures S1, S2 and S3.

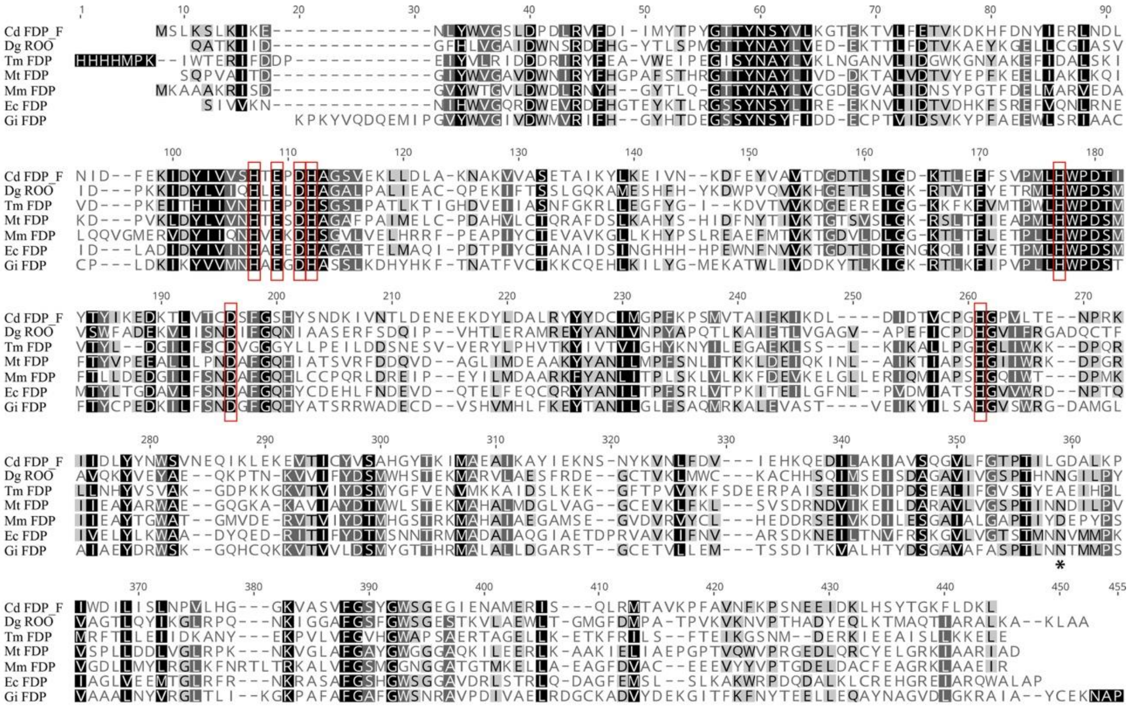

Figure S1 – Amino acid sequence alignment of flavodiiron proteins cores

The amino acid sequence alignment was based on structural superpositions of FDPs available structures using Modeller [1]. Then, ClustalX [2] in Profile Alignment Mode was used to align the sequence of the *C. difficile* FDP_F core domains (residues 1-400). Amino acid residues that coordinate the catalytic iron atoms are highlighted with red boxes. Strictly conserved amino acids represented as black boxes, whereas dark grey boxes represent the mostly conserved residues among the selected sequences. Besides *C. difficile* FDP_F core, the sequences represented in the alignment are the following: Dg ROO, *Desulfovibrio gigas* Rubredoxin Oxygen:oxidoreductase (PDB 1E5D); Tm FDP, *Thermotoga maritima* (PDB 1VME); Mt FDP, *Moorella thermoacetica* (PDB 1YCF); Mm, *Methanothermobacter marburgensis* (PDB 2OHH); Ec FDP, *Escherichia coli* (PDB 4DO2) and Gi FDP, *Giardia intestinalis* (PDB 2Q9U).

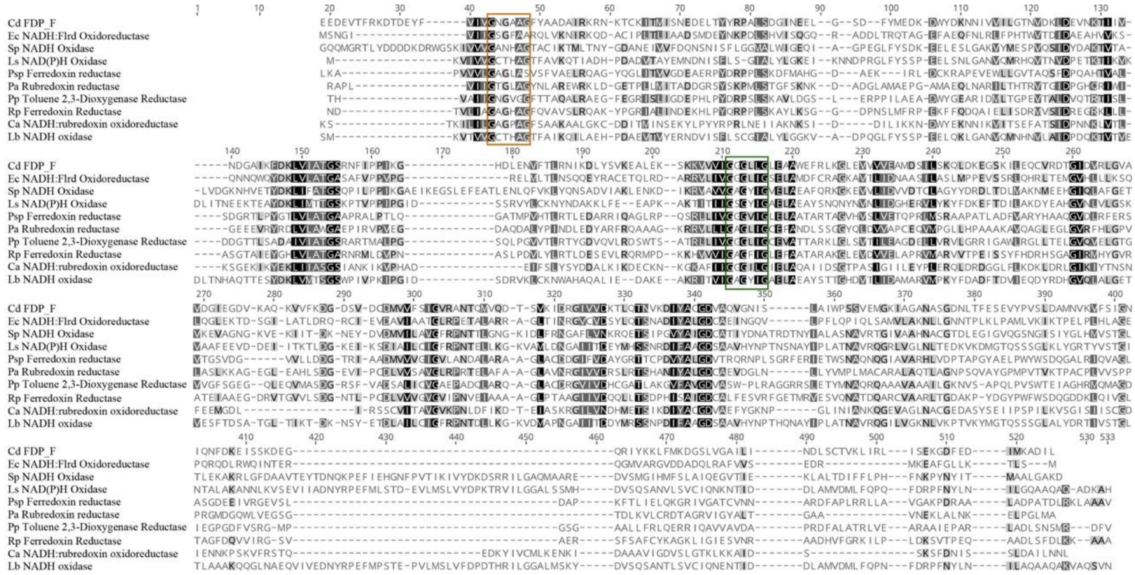

Figure S2 – Amino acid sequence alignment of NADH:Rubredoxin oxidoreductase domain of C. difficile FDP_F. The amino acid sequence alignment was based on structural superpositions using Modeller [1] of the top hits obtained through prediction of the structure of the FDP_F C-terminal oxidoreductase domain using Phyre2.0 [3]. Then, ClustalX [2] in Profile Alignment Mode was used to align the sequences of the *C. difficile* FDP_F oxidoreductase domain (residues 445 to 843) and the E. coli NADH:flavorubredoxin oxidoreductase. The Glycine reach domains proposed to bind FAD and NADH are highlighted in brown and green boxes respectively. Amino acid residues strictly conserved are represented as black boxes, whereas dark grey boxes represent the mostly conserved residues among the selected sequences. Besides *C. difficile* FDP_F oxidoreductase domain and and the E. coli NADH:flavorubredoxin oxidoreductase, the sequences represented in the alignment are the following, *Streptococcus pyogenes* (Sp) NADH oxidase (PDB 2BC0), *Lactobacillus sanfranciscensis* (Ls) NAD(P)H oxidase (PDB 2CDU), *Pseudomonas sp.* (Psp) (strain KKS102) ferredoxin reductase (PDB 2GR2), *Pseudomonas aeruginosa* (Pa) rubredoxin reductase (PDB 2V3A), *Pseudomonas putida* (Pp) toluene 2,3-dioxygenase reductase (PDB 3EF6), *Rhodopseudomonas palustris* (Rp) ferredoxin reductase (PDB 3FG2), *Clostridium acetobutylicum* (Ca) NADH:rubredoxin oxidoreductase (PDB 3KLJ) and *Lactobacillus brevis* (Lb) NADH oxidase (PDB 5ER0).

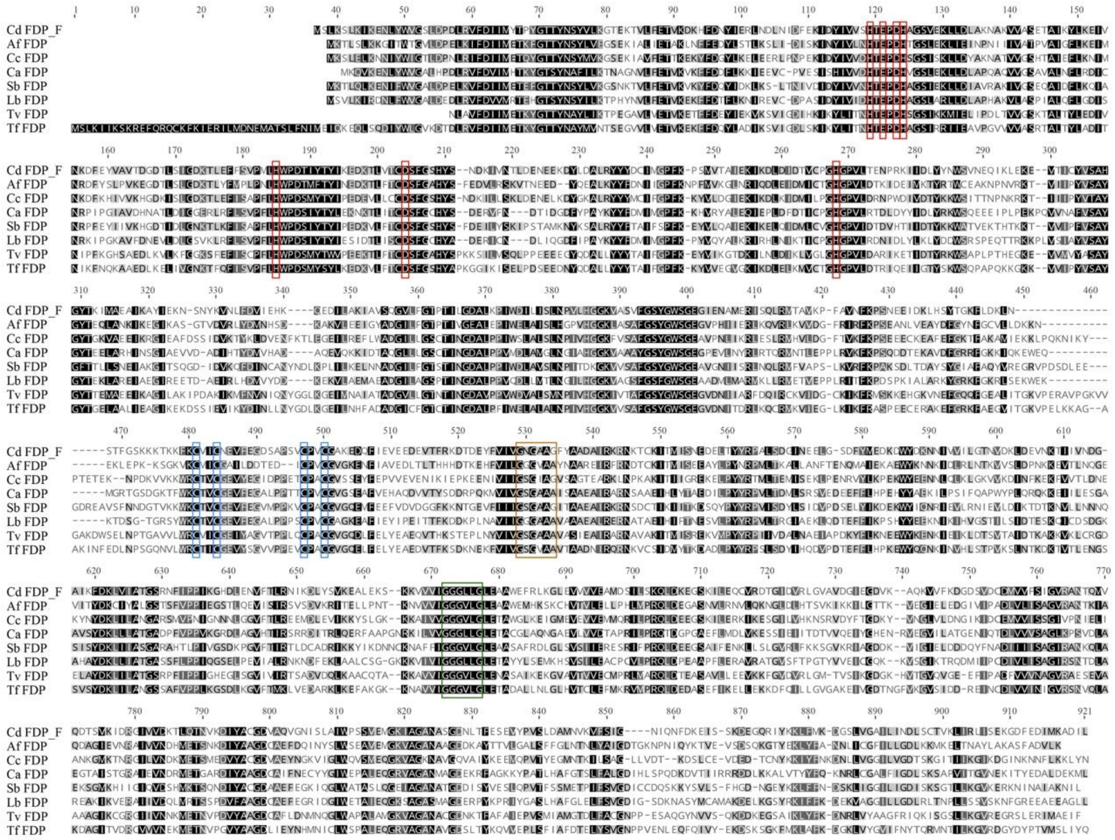

Figure S3 – Amino acid sequence alignment of Class F FDPs from selected phyla. The amino acid sequence alignment was performed using ClustalX [2], for sequences from organisms belonging to distinct phyla. Amino acid residues that coordinate the catalytic diiron atoms are highlighted with red boxes while the amino acids that coordinate the rubredoxin center are highlighted in blue boxes. Glycine reach domains proposed to bind FAD and NADH are highlighted in brown and green boxes respectively. Amino acid residues strictly conserved are represented as black boxes, whereas dark grey boxes represent the mostly conserved residues among the selected sequences. Besides *C. difficile* FDP_F (Cd), the sequences from other Class F FDPS are from the following organisms: *Anaerorhabdus furcosa* (Af), *Cetobacterium ceti* (Cc), *Chitinivibrio alkaliphilus* (Ca), *Spirochaetes bacterium* (Sb), *Lentisphaerae bacterium* (Lb), *Trichomonas vaginalis* (Tv) and *Tritrichomonas foetus* (Tf).

References

1 Webb, B. & Sali, A. Comparative Protein Structure Modeling Using MODELLER. *Current Protocols in Bioinformatics* **54**, 5.6.1-5.6.37, doi:doi:10.1002/cpbi.3 (2016).

2 Larkin, M. A. *et al.* Clustal W and clustal X version 2.0. *Bioinformatics* **23**, 2947-2948, doi:10.1093/bioinformatics/btm404 (2007).

3 Kelley, L. A., Mezulis, S., Yates, C. M., Wass, M. N. & Sternberg, M. J. E. The Phyre2 web portal for protein modeling, prediction and analysis. *Nat Protoc* **10**, 845-858, doi:10.1038/nprot.2015.053 (2015).
